# In Vitro Study on the Efficacy of *Ziziphus vulgaris* L. and *Camellia sinensis* (L.) Kuntze Extracts for Controlling *Varroa destructor* (Acari: Varroidae) in *Apis mellifera* (Hym.: Apidae)

**DOI:** 10.1002/vms3.70612

**Published:** 2025-09-13

**Authors:** Salim Ojagh, Farnaz Malekifard, Mohammad Yakhchali

**Affiliations:** ^1^ Department of Pathobiology Faculty of Veterinary Medicine Urmia University Urmia Iran

**Keywords:** *Camellia sinensis* (L.) Kuntze, *Varroa destructor*, *Ziziphus vulgaris* L, *in vitro*

## Abstract

**Background:**

*Ziziphus vulgaris* L. and *Camellia sinensis* (L.) Kuntze are medicinal plants widely used in various ethnomedical systems, particularly in Iranian Traditional Medicine, for the treatment of various diseases.

**Objectives:**

While their biological activities are well‐documented, their effectiveness against *Varroa destructor*, a significant ectoparasite of *Apis mellifera* (honeybees), remains unknown. As *V. destructor* poses a serious threat to honeybee populations globally, it is crucial for researchers to explore novel control methods.

**Methods:**

In this study, we evaluated the anti‐*Varroa* activity of *Z. vulgaris* and *C. sinensis* at different concentrations (5, 10, 15, 20 and 25 µL/L air) and exposure times (5, 10, 15, 20 and 25 h).

**Results:**

The results showed that mite mortality increased in parallel with the increase in extract concentration and exposure time. Importantly, the plant extracts did not cause significant mortality in *A. mellifera* compared to the control group.

**Conclusions:**

In conclusion, our findings suggest that these plant extracts could be effective agents for managing *V. destructor*.

## Graphical abstract

Extracts of *Ziziphus vulgaris* and *Camellia sinensis* applied in wooden cages increase *Varroa destructor* mortality (5–25 µL/L air) while minimizing *Apis mellifera* harm, offering a sustainable mite control method.

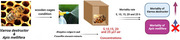

## 1 Introduction

*Varroa destructor* (Anderson and Trueman 2000), formerly *Varroa jacobsoni*, was first identified in 1949 and primarily attacks honeybees, posing a major threat to their populations globally. This parasitic mite has two growth phases: the phoretic phase, where it feeds on adult bees, and a reproductive phase targeting larvae and pupae (Rosenkranz et al. 2010).

Synthetic acaricides, including pyrethroids and organophosphates, are widely used in commercial beekeeping, but resistance has developed in various mite strains (Bahreini et al. 2025; Bertola and Mutinelli 2025; Marsky et al. 2024), complicating control efforts. In addition, these chemicals may cause persistent environmental contamination (Rosenkranz et al. 2010), prompting research into novel organic pesticides with lower environmental impact for integrated pest management (Maggi et al. 2010; Karimi et al. 2022). Studies have shown that extracts from certain plants can effectively control *Varroa* mites without causing harmful effects (Karimi et al. 2022; Ramezanipour and Yakhchali 2020).

Green tea, derived from dried leaves of *Camellia sinensis*, contains a number of physiologically active substances, including polyphenols, methylphenols, essential oils, proteins, vitamins and amino acids (Yamamoto et al. 1997). Previous research has focused on extracting polyphenols from *C. sinensis* (Perva‐Uzunalić et al. 2006; Senanayake 2013) and assessing their potential antioxidant, antiviral and anticancer properties (Adhami and Mukhtar 2007; Dai and Mumper 2010; Feitelson et al. 2015; Yang et al. 2019). The most common ingredient found in *C. sinensis* is catechins, which are primarily responsible for their biological properties (Benelli et al. 2002). These benefits include lowering plasma lipids, reduction of inflammation and the development of antibacterial, antimalarial, anticancer and antioxidant properties (Bohm 1998; Huang et al. 1998; Vijaya et al. 1995). Catechins, which belong to the flavan‐3‐ol family, have attracted considerable interest because of their potential therapeutic effects. Strong antioxidants and antiviral properties may help to protect against the harmful effects of the disease (Katada et al. 2020; Sanlier et al. 2018; Yang et al. 2019). For example, the study by Paveto et al. demonstrated that green tea catechins have an effective therapeutic activity against *Trypanosoma cruzi* in vitro, indicating that they may be useful in the treatment of Chagas disease (Paveto et al. 2004).

*Ziziphus vulgaris*, commonly known as jujube, is a member of the Rhamnaceae family. The plant is widely grown in regions such as Burma, Iran and various parts of India (Goli‐malekabadi et al. 2014). Phytochemical analysis shows that *Z. vulgaris* contains bioactive compounds, including cyclopeptide alkaloids. Several studies suggest that these cyclopeptide alkaloids contribute to the antiplasmodial and antimycobacterial properties of the plant. In addition, *Ziziphus* contains terpenoids, alkaloids, steroids, polysaccharides and glycosides of the saponin family (Dodangeh et al. 2017; Panseeta et al. 2011). The research by Rahimi et al. (2024) showed that *Z. vulgaris* extract shows anti‐*Trichomonas gallinae* activity in vitro. Jujube is therefore considered a promising candidate for the development of new antiparasitic drugs.

This study assessed the varroacidal effects of extracts from *C. sinensis* and *Z. vulgaris*. The aim of this study was to assess the effects of these extracts on *Varroa* mites in wooden cages. In addition, the study also looked at mortality rates of *Apis mellifera* after in vitro treatment.

## 2 Material and Methods

### 2.1 Preparation of Plant Extract

The plants *Z. vulgaris* and *C. sinensis* were bought from the Persian herbal market and certified by the University of Agriculture of Urmia in Iran. It should be noted that these extracts have also been used in our previous study (Rahimi et al. 2024). The technique used by Baqer et al. was modified in our process and the herbal extracts were prepared accordingly. All dried plant material, including green tea leaves and dried fruit of the jujube, was placed in an electric mixer (Moulinex, Paris, France) and ground to a fine powder. We mixed 100 g of powdered plant material with 500 mL of 70% ethanol and stirred the solution with a magnetic stirrer for 2 h. Afterwards, the solution was left unchanged at room temperature for 24 h. It was stirred again before it was filtered. The solvent was then removed by means of a rotating evaporator. For future applications, the semi‐solid residual components were stored at 4°C to maintain stability and subsequently freeze‐dried (Baqer et al. 2014; Rahimi et al. 2024).

### 2.2 Gas Chromatography‐Mass Spectrometry Analysis

Gas chromatography‐mass spectrometry (GC‐MS) (Thermo Scientific, Paris, France) was utilized to analyse the chemical composition of the extract. The split ratio for the helium carrier gas was set at 0.50 mL/min. The gas chromatography conditions included an initial temperature of 40°C, ramping to 250°C at a rate of 80°C/min, with injector and detector temperatures maintained at 250°C. Individual compounds were identified by comparing their relative retention times on a capillary column with those of authentic samples, as well as by matching their peak‐to‐peak mass spectra to published data (Khoshnejad et al. 2023).

### 2.3 Fumigant Toxicity Tests and Survival Rate

The fumigant toxicity of the extracts on *V. destructor* and *A. mellifera* was assessed by introducing 100 adult honeybees, taken from heavily infested hives containing approximately 30% *Varroa* mite infection, into a wooden cage measuring 14 × 12 × 9 cm. An 8‐mesh plastic screen was positioned about 2 cm above the bottom of the cage to separate the dead honeybees from the fallen mites. To trap the fallen mites, a Vaseline‐coated paper was placed on the cage floor (Ghasemi et al. 2016). The treatment process for the infected honeybees began with staining filter papers using varying concentrations (5, 10, 15, 20 and 25 µL/L air) of extracts, applied without any solvent. The treated filter paper was placed on top of the cage to facilitate volatilization. It is important to note that the number of mites on each honeybee was consistent throughout all phases of the experiment, with 15 adult mites present on each bee. The number of mites on each honeybee was standardized at 15 adult mites per bee, achieved by manually transferring mites from heavily infested hives (30% infection rate) using fine forceps under a stereomicroscope (Ghasemi et al. 2016). Five exposure periods (5, 10, 15, 20 and 25 h) were selected for exposing mites and honeybees to the extracts. A 50% sugar syrup was provided as a food source for the honeybees during the test. The treatments were conducted in darkness at a temperature of 32°C and a relative humidity of 70%. Control groups were maintained under the same conditions, but no extracts were applied to these control cages. A completely randomized design was employed for the experiment, and each combination of concentration and exposure time was replicated three times. After the incubation period, the total number of dead mites (fallen onto Vaseline‐coated paper) and surviving mites (remaining on bees or in the cage) were counted to determine the overall mortality percentage. For accuracy, dead mites with damage to pedipalps, legs, or idiosoma were excluded from the mortality data to avoid confounding effects from mechanical injury (Ghasemi et al. 2016). In the final stage of analysis, living honeybees were collected to isolate and count any surviving mites remaining on them.

### 2.4 Statistical Analysis

SPPS software (ver. 26.0) was used for statistical analysis and ANOVA was used for analysis of variance. The data were presented as the mean ± SD (standard deviation), and the values lower than 0.05 (*p* < 0.05) were considered significant.

## 3 Results

### 3.1 Gas Chromatography‐Mass Spectrometry Analysis

Analysis using GC‐MS identified the following key chemical components in the studied extracts: In the extract of *Z. vulgaris*, the prominent compounds included catechin (22.67%), quercitrin (19.83%), Kaempferol (14.44%) and quinic acid (11.45%) as shown in Table 1. In the extract of *C. sinensis*, the most significant components were methyl linoleate (24.07%), naphthalene (12/36%), squalene (11.34%) and *n*‐hexadecanoic acid (9.32%), also detailed in Table 1.

**TABLE 1:** Major chemical compounds in *Ziziphus vulgaris* L. and *Camellia sinensis* (L.) Kuntze extracts identified by GC‐MS.

Extracts	Major compounds	Percent
*Ziziphus vulgaris*	Quinic acid	11.45
	Gallic acid	8.74
	Catechin	22.67
	Epicatechin	2.08
	Quercitrin	19.83
	Naringin	5.34
	Acacetin	2.13
	Kaempferol	14.44
	3,4‐di‐*O*‐caffeoylquinic acid	1.22
	1,3‐di‐*O*‐caffeoylquinic acid	4.56
*Camellia sinensis*	Squalene	11.34
	Methyl stearate	3.05
	Methyl linolenate	7.43
	Methyl linoleate	24.07
	Naphthalene	12.36
	α‐bulnesene	2.52
	Eremophilene	1.22
	γ‐muurolene	1.38
	*n*‐hexadecanoic acid	9.32
	Methyl hexadecanoate	0.43

### 3.2 Assessment of Survival Rate

The dose‐dependent mortality trends of *V. destructor* and *A. mellifera* after exposure of 5, 10, 15, 20 and 25 h under cage conditions are presented in Tables 2 and 3. Table 2 shows significant variations in the susceptibility of *V. destructor* to different concentrations of the extracts. Specifically, the mortality rate of *V. destructor* exposed to 25 µL/L air concentrations of *Z. vulgaris* extract and *C. sinensis* extract for 15 h reached 100%. In contrast, as indicated in Table 3, the mortality rate of *A. mellifera* at these same concentrations was less than 2%.

**TABLE 2:** Mortality percentage (mean ± SD) of *Varroa destructor* after exposure to different concentrations of *Ziziphus vulgaris* L. and *Camellia sinensis* (L.) Kuntze extracts under cage conditions.

Groups	5 h	10 h	15 h	20 h	25 h
Control (‐)	00.00 ± 0.0^Eb^	00.00 ± 0.0^Hb^	1.43 ± 0.06^Gb^	1.98 ± 0.11^Eb^	4.03 ± 0.08^Ca^
*Ziziphus vulgaris*					
5 µL/L air	1.02 ± 0.08^Ee^	14.75 ± 0.30^Gd^	31.09 ± 1.34^Fc^	60.44 ± 1.09^Db^	89.87 ± 2.44^Ba^
10 µL/L air	9.67 ± 0.83^De^	30.22 ± 2.42^Ed^	42.67 ± 2.55^Ec^	71.08 ± 2.77^Cb^	100.00 ± 0.0^Aa^
15 µL/L air	18.54 ± 1.35^Cd^	49.57 ± 1.88^Dc^	79.54 ± 2.08^Cb^	90.32 ± 0.45^Ba^	100.00 ± 0.0^Aa^
20 µL/L air	32.06 ± 1.88^Bc^	79.44 ± 0.32^Bb^	97.67 ± 2.45^Aa^	100.00 ± 0.0^Aa^	100.00 ± 0.0^Aa^
25 µL/L air	41.08 ± 2.65^Ac^	88.67 ± 2.45^Ab^	100.00 ± 0.0^Aa^	100.00 ± 0.0^Aa^	100.00 ± 0.0^Aa^
*Camellia sinensis*					
5 µL/L air	1.17 ± 0.12^Ee^	19.43 ± 2.77^Fd^	39.66 ± 1.87^EFc^	75.21 ± 2.82^Cb^	89.23 ± 2.44^Ba^
10 µL/L air	7.18 ± 0.75^De^	34.22 ± 3.07^Ed^	53.66 ± 2.89^Dc^	84.78 ± 1.47^Bb^	99.07 ± 3.54^Aa^
15 µL/L air	20.66 ± 2.34^Cd^	69.03 ± 1.68^Cc^	89.89 ± 1.05^Bb^	100.00 ± 0.0^Aa^	100.00 ± 0.0^Aa^
20 µL/L air	31.66 ± 2.05^Bc^	80.79 ± 2.07^Bb^	97.11 ± 1.07^Aa^	100.00 ± 0.0^Aa^	100.00 ± 0.0^Aa^
25 µL/L air	35.44 ± 1.67^Bc^	89.04 ± 1.74^Ab^	100.00 ± 0.0^Aa^	100.00 ± 0.0^Aa^	100.00 ± 0.0^Aa^

*Note*: Different superscripts (A–H) within the same column indicate significant toxicity effects of different concentrations of extracts within each exposure time. Different superscripts (a–e) within the same row indicate significant toxicity effects of each concentration of extracts during the different exposure times.

**TABLE 3:** Mortality percentage (mean ± SD) of *Apis mellifera* after exposure to different concentrations of *Ziziphus vulgaris* L. and *Camellia sinensis* (L.) Kuntze extracts under cage conditions.

Groups	5 h	10 h	15 h	20 h	25 h
Control (‐)	00.00 ± 0.0^Aa^	00.00 ± 0.0^Aa^	00.00 ± 0.0^Aa^	0.10 ± 0.00^Aa^	0.40 ± 0.07^Aa^
*Ziziphus vulgaris*					
5 µL/L air	00.00 ± 0.0^Aa^	00.00 ± 0.0^Aa^	00.00 ± 0.0^Aa^	0.24 ± 0.03^Aa^	0.58 ± 0.04^Aa^
10 µL/L air	00.00 ± 0.0^Aa^	00.00 ± 0.0^Aa^	00.00 ± 0.0^Aa^	0.63 ± 0.08^Aa^	1.02 ± 0.13^Aa^
15 µL/L air	00.00 ± 0.0^Aa^	00.00 ± 0.0^Aa^	1.05 ± 0.16^Aa^	1.25 ± 0.11^Aa^	1.30 ± 0.11^Aa^
20 µL/L air	00.00 ± 0.0^Aa^	00.00 ± 0.0^Aa^	1.18 ± 0.14^Aa^	1.41 ± 0.18^Aa^	1.52 ± 0.22^Aa^
25 µL/L air	00.00 ± 0.0^Aa^	00.00 ± 0.0^Aa^	1.55 ± 0.11^Aa^	1.60 ± 0.06^Aa^	1.69 ± 0.14^Aa^
*Camellia sinensis*					
5 µL/L air	00.00 ± 0.0^Aa^	00.00 ± 0.0^Aa^	00.00 ± 0.0^Aa^	00.00 ± 0.0^Aa^	0.59 ± 0.10^Aa^
10 µL/L air	00.00 ± 0.0^Aa^	00.00 ± 0.0^Aa^	0.02 ± 0.01^Aa^	0.52 ± 0.03^Aa^	0.61 ± 0.12^Aa^
15 µL/L air	00.00 ± 0.0^Aa^	00.00 ± 0.0^Aa^	1.06 ± 0.08^Aa^	1.25 ± 0.04^Aa^	1.03 ± 0.15^Aa^
20 µL/L air	00.00 ± 0.0^Aa^	00.00 ± 0.0^Aa^	1.12 ± 0.15^Aa^	1.34 ± 0.13^Aa^	1.45 ± 0.09^Aa^
25 µL/L air	00.00 ± 0.0^Aa^	00.00 ± 0.0^Aa^	1.03 ± 0.13^Aa^	1.54 ± 0.10^Aa^	1.76 ± 0.21^Aa^

*Note*: Different superscripts within the same column indicate significant toxicity effects of different concentrations of extracts within each exposure time. Different superscripts within the same row indicate significant toxicity effects of each extract concentration at different exposure times.

Although various concentrations of the extracts demonstrated acaricidal effects against *V. destructor*, they exhibited low toxicity to *A. mellifera*. As shown in Table 2, the acaricidal activity of *Z. vulgaris* extract at concentrations of 10, 15, 20 and 25 µL/L air and *C. sinensis* extract at 15, 20 and 25 µL/L air resulted in 100% mortality of *V. destructor after* 25 h of exposure. However, as Table 3 illustrates, the mortality rate of *A. mellifera* remained below 2%.

## 4 Discussion

Herbal therapies are popular treatments because they are natural, affordable and environmentally friendly. Recent studies have shown that plant extracts may be as effective as or more effective than conventional acaricides (Choi et al. 2004). Our study assessed the mortality rates of *V. destructor* and *A. mellifera* after exposure to extracts of *C. sinensis* and *Z. vulgaris*.

The results of our study indicated that exposure to *C. sinensis* and *Z. vulgaris* extracts increased the mortality rate of *V. destructor*. Numerous studies have assessed the effectiveness of various plants against *Varroa* mites. For example, Ramezanipour and Yakhchali (2020) investigated the impact of a hydroalcoholic extract from *Ferula pseudalliacea* (Family: Apiaceae) on *Varroa* infestation in honeybees, finding that the extract had a lethal effect on *V. destractor*. Another study by Schenk et al. (2001) demonstrated the dose‐dependent effects of *Azadirachta indica* L. (neem oil). In Iran, Karimi et al. (2022) reported the varroacidal effects of *Melissa officinalis* L., *Quercus infectoria* G. Olivier and *Ceratonia siliqua* L. essential oils. In addition, Ariana et al. (2002) highlighted the toxic effects of essential oils derived from *Satureja hortensis* L., *Zataria multiflora*, *Mentha spicata* L., *Rosmarinus officinalis* L., *Origanum vulgare* L., *Lavandula officinalis* L. and *Anethum graveolens* L. on *Varroa* mites. Imdorf et al. (2006) demonstrated the effectiveness of *Thymus* (*Thymus vulgaris* L.), *Salvia* (*Salvia officinalis* L.) and *Hyssop* (*Hyssopus officinalis* L.) against *V. destructor*. Fassbinder et al. (2002) found that treating colonies infested with *Varroa* using monoterpenoids—such as (*R*)‐myrtenyl acetate, linalyl acetate, thymol acetate and (*S*)‐perillyl acetate significantly reduced *V. destructor* populations. Furthermore, Ruffinengo et al. (2005) studied the biological effects of Argentinian wild plant species on *V. destructor*, revealing that *Acantholippia seriphioides* mold oil showed the highest potential for mite control. Lastly, a study by Bava et al. (2021) reported the efficacy of essential oils from five different species of *Citrus* spp. in managing *Varroa* infestations.

Our study found that *C. sinensis* could increase the mortality rate of the *Varroa* mite. In addition, we demonstrated that the pesticidal effects of the extract might be linked to its main constituents. In this study, caffeine (24.07%) was identified as the primary component of the *C. sinensis* extract, which has been reported to have antiparasitic effects in several studies. These studies indicated that both *C. sinensis* extract and caffeine exhibited anti‐*Acanthamoeba*, anti‐*T. gallinae* and antileishmanial effects (Hajihossein et al. 2020; Rahimi et al. 2024; Tadesse et al. 2015). Previous research (Parvez et al. 2019) has shown that *C. sinensis* and its constituents displayed antimicrobial activity against multidrug‐resistant gram‐positive and gram‐negative bacteria. Moreover, *C. sinensis* was effective in inhibiting *Leishmania amazonensis* (dos Reis et al. 2013; Inacio et al. 2013), reducing exposure to *Haemonchus contortus* worms (Zhong et al. 2014) and inhibiting both the promastigote and amastigote forms of *Leishmania braziliensis* (Inacio et al. 2014). In addition, *C. sinensis* was found to inhibit *Babesia* spp., *Eimeria* spp., *T. gallinae* and *T. cruzi* (Aboulaila et al. 2010; Jang et al. 2007; Rahimi et al. 2024). Research conducted by Fakae et al. demonstrated that beers made from *C. sinensis* exhibited amoebic activity against *Acanthamoeba* trophozoites and effectively prevented the parasite from encysting. Their findings suggested that *C. sinensis* could serve as a source of inhibitors for the growth and encystation of *Acanthamoeba castellanii* (Fakae et al. 2020).

The present study utilized *Z. vulgaris* to evaluate the mortality rate of *Varroa* mites. Our findings indicated that increasing the concentration of the extract correspondingly increased the mortality percentage of the *Varroa* mite. This aligns with the results from a study conducted by Rahimi et al., which observed a dose‐ and time‐dependent anti‐*T. gallinae* effect of *Z. vulgaris* extract (Rahimi et al. 2024). The higher efficacy of *Z. vulgaris* on adult ticks may be attributed to its chemical composition. In our study, we identified catechin (22.67%) as one of the components in the jujube extract, along with smaller amounts of other compounds. Previous studies have also demonstrated the antiparasitic properties of catechin, quercitrin and Kaempferol, which are the primary components found in jujube in this research (Calzada 2005; Dodson et al. 2011). In addition, our results support earlier investigations into the antiparasitic, antifungal and antimicrobial properties of *Z. vulgaris* cyclopeptide alkaloids, tannins and saponins (Chung et al. 1998; Dodangeh et al. 2017; Panseeta et al. 2011). In our in vitro study after adding the jujube extract (25 µL/L air), we observed that mites were no longer present after 15 h. Other research has confirmed that jujube extract is effective in vitro against *Acanthamoeba* spp. and malaria (Panseeta et al. 2011). The amoebicidal effect of *Z. vulgaris* is linked to its ability to induce apoptosis, as reported by Dodangeh et al. (2017). In addition, previous research highlighted that inducing apoptosis with *Z. vulgaris* offers significant advantages over other commonly used drugs, such as metronidazole.

## 5 Conclusions

The results of this study indicate that *C. sinensis* and *Z. vulgaris* extracts could serve as novel anti‐*Varroa* agents because of their antiparasitic properties. In addition, these extracts have medicinal uses and can help mitigate the harmful effects of pesticides on both the environment and human health. However, Further research is needed to assess residue levels in bee products (e.g., honey, wax) and develop formulations to minimize contamination risks in field applications, ensuring safety for consumers.

## Author Contributions

**Salim Ojagh**: conceptualization, investigation, funding acquisition, methodology, writing – review and editing, software, data curation, resources. **Farnaz Malekifard**: conceptualization, investigation, funding acquisition, writing – original draft, methodology, validation, visualization, writing – review and editing, software, formal analysis, project administration, data curation, supervision, resources. **Mohammad Yakhchali**: conceptualization, writing – original draft, validation, methodology, project administration, data curation, supervision, resources.

## Ethics Statement

The authors confirm the ethical policies of the Journal, as noted on the journal's author guidelines page. The study was carried out following the regulations of the Animal Ethics Committee of the Urmia University, Urmia, Iran (IR‐UU‐AEC‐3/6).

## Conflicts of Interest

The authors declare no conflicts of interest.

## Data Availability

The data that support the findings of this study are available on request from the corresponding author. The data are not publicly available due to privacy or ethical restrictions.
